# 伴t(14;16)的初治多发性骨髓瘤19例预后分析

**DOI:** 10.3760/cma.j.cn121090-20240814-00303

**Published:** 2024-10

**Authors:** 叶 李, 俊茹 刘, 娟 李, 文明 陈

**Affiliations:** 1 首都医科大学附属北京朝阳医院血液科 北京市多发性骨髓瘤医疗研究中心，北京 100020 Department of Hematology, Beijing Chaoyang Hospital, Capital Medical University, Myeloma Research Center of Beijing, Beijing 100020, China; 2 北京大学人民医院，北京大学血液病研究所，国家血液系统疾病临床医学研究中心，造血干细胞移植治疗血液病北京市重点实验室，北京 100044 Peking University People's Hospital, Peking University Institute of Hematology, National Clinical Research Center for Hematologic Disease, Beijing Key Laboratory of Hematopoietic Stem Cell Transplantation for Hematological Diseases, Beijing 100044, China; 3 中山大学附属第一医院血液科，广州 510080 Department of Hematology, The First Affiliated Hospital of Sun Yat-Sen University, Guangzhou 510080, China

**Keywords:** 多发性骨髓瘤, 预后, t（14;16）, 细胞遗传学, Multiple myeloma, Prognosis, t（14;16）, Cytogenetics

## Abstract

**目的:**

探讨伴t（14;16）初治多发性骨髓瘤（MM）患者的预后。

**方法:**

收集2018年1月至2020年11月首都医科大学附属北京朝阳医院和中山大学附属第一医院564例初治MM患者的临床资料并进行回顾性分析，比较伴t（14;16）患者与FISH正常、伴t（4;14）、伴del（17p）患者的预后。

**结果:**

564例初治MM患者中19例（3.4％）伴t（14;16）异常，其中14例合并1q21+，3例合并del（17p）。与FISH正常的患者相比，t（14;16）患者具有较短的无进展生存（PFS）期和总生存（OS）期（中位PFS期分别为14个月和未达到，*P*<0.001；中位OS期分别为42个月和未达到，*P*＝0.002）。倾向性评分匹配后的15例伴t（14;16）和15例伴t（4;14）患者PFS期和OS期的差异均无统计学意义（中位PFS期分别为13.0个月和未达到，*P*＝0.247；中位OS期分别为42个月和未达到，*P*＝0.609）。倾向性评分匹配后的15例伴t（14;16）和15例伴del（17p）患者PFS期和OS期的差异均无统计学意义（中位PFS期分别为13个月和31个月，*P*＝0.939；中位OS期分别为42个月和37.3个月，*P*＝0.557）。同时合并1q21+时，倾向性评分匹配后的伴t（14;16）患者与伴t（4;14）、伴del（17p）患者PFS期和OS期的差异均无统计学意义（*P*值均>0.05）。伴t（14;16）患者是否进行auto-HSCT对PFS和OS均无明显影响（*P*值均>0.05）。

**结论:**

在初治MM患者中，t（14;16）常伴随其他高危细胞遗传学异常出现，其不良预后价值与t（4;14）及del（17p）相似。

## Abstract (en)

**Objective:**

To investigate the prognosis of newly diagnosed multiple myeloma（MM）patients with t (14;16）abnormality.

**Methods:**

Clinical data from 564 patients diagnosed with initial MM from January 2018 to November 2020 at Beijing Chaoyang Hospital affiliated with Capital Medical University and the First Affiliated Hospital of Sun Yat-sen University were collected and retrospectively analyzed. The prognoses of patients with t（14;16）were analyzed and compared with the prognoses of patients with normal FISH, and those with t（4;14）and del（17p）.

**Results:**

Among 564 newly diagnosed MM patients, 19（3.4%）exhibited t（14;16）abnormalities, with 14 cases diagnosed with 1q21+ and three cases with del（17p）. Progression-free survival（PFS）and overall survival（OS）of patients with t（14;16）were significantly shorter compared with patients with normal FISH（the median PFS: 14 months *vs* not reached, *P*<0.001; the median OS: 42 months *vs* not reached, *P*＝0.002）. No statistically significant difference was detected in PFS and OS between the 15 patients with t（14;16）and the 15 with t（4;14）after propensity score matching（the median PFS: 13.0 months *vs* not reached, *P*＝0.247; the median OS: 42 months *vs* not reached, *P*＝0.609）. Similarly, no statistically significant difference was observed in PFS and OS between 15 patients with t（14;16）and 15 with del（17p）（the median PFS: 13 months *vs* 31 months, *P*＝0.939; the median OS: 42 months *vs* 37.3 months, *P*＝0.557）. Propensity score matching indicated that when combined with 1q21+, no statistically significant differences were present in PFS and OS between patients with t（14;16）and patients with t（4;14）or patients with del（17p）（all *P*>0.05）. Whether or not the patients with t（14;16）had undergone auto-HSCT did not significantly impact the PFS and OS（all *P*>0.05）.

**Conclusion:**

t（14;16）is often associated with high-risk cytogenetic abnormalities in newly diagnosed MM patients, and its adverse prognostic value is similar to that of t（4;14）and del（17p）.

多发性骨髓瘤（MM）是一种具有复杂细胞遗传学特征的骨髓浆细胞克隆性增殖的血液系统恶性肿瘤[1]。t（14;16）（q32;q23）是一种少见的原发性细胞遗传学异常，见于约4％的初治MM患者[2]–[4]，主要涉及14号染色体的IGH位点和16号染色体的MAF位点，能够引起致癌转录因子c-MAF的过度表达[5]。修订版国际分期系统（R-ISS）把t（14;16）、t（4;14）和del（17p）定义为高危预后因素[6]。mSMART3.0[1]及2016版的国际骨髓瘤工作组（IMWG）指南[7]同样把t（14;16）归为高危MM异常。2009版的IMWG指南把t（4;14）及del（17p）归为高危细胞遗传学异常（HRCA），t（14;16）因为标本量少未作为预后分层指标纳入[8]。同样，2022年欧洲骨髓瘤工作组制定的R2-ISS将ISS Ⅲ期（1.5分）、ISS Ⅱ期（1分）、del（17p）（1分）、LDH升高（1分）、t（4;14）（1分）及1q21+（0.5分）纳入预后评分系统[9]，由于多因素分析中总生存（OS）的差异有统计学意义，而无进展生存（PFS）的差异无统计学意义（*HR*＝1.15，95％ *CI* 0.96～1.37, *P*＝0.13），未把t（14;16）作为具有独立预后意义的危险因素纳入其中。既往文献报道的伴t（14;16）患者的数据较少，预后价值不明确。本文回顾性分析了首都医科大学附属北京朝阳医院和中山大学附属第一医院的临床数据，旨在探索t（14;16）的预后价值。

## 病例与方法

1. 病例：本项研究回顾性纳入2018年1月至2020年11月在首都医科大学附属北京朝阳医院和中山大学附属第一医院住院治疗的564例具有完整FISH结果和随访记录的初治MM患者，其中19例（3.4％）伴t（14;16）异常，109例（19.3％）伴t（4;14）异常、55例（9.8％）伴del（17p）异常。收集的临床基线特征包括性别、年龄、HGB、肌酐、血钙、白蛋白、LDH、β_2_-微球蛋白（β_2_-MG）、M蛋白类型、ISS分期和治疗情况。为减少基线治疗的不平衡，将19例伴t（14;16）患者分别与109例伴t（4;14）和55例伴del（17p）患者按照1∶1的比例进行倾向性评分匹配（PSM）分析。PSM的变量包括性别、年龄、HGB、肌酐、血钙、白蛋白、LDH、β_2_-MG、M蛋白类型、ISS分期、伴1q21+、伴del（17p）、伴t（4;14）、诱导治疗方案及是否进行自体造血干细胞移植（auto-HSCT）。所有患者的诊断均依据IMWG的MM诊断标准。该研究获得首都医科大学附属北京朝阳医院伦理委员会批准（伦理批号：2022-科-493）。

2. 诱导治疗：所有患者均接受至少含一种新药的诱导治疗方案，包括以蛋白酶体抑制剂（PI）为基础的方案、以免疫调节药物（IMiD）为基础的方案及PI联合IMiD方案。适合移植的患者先接受4个疗程的诱导治疗，然后进行auto-HSCT巩固治疗，auto-HSCT后3个月应用来那度胺等药物维持治疗。所有MM患者疗效的评估均依据IMWG标准。

3. FISH检测：所有样本均采用骨髓标本，经CD138磁珠分选富集后进行荧光原位杂交（FISH）检测。应用的FISH探针包括TP53（17p13.1）、1q21（1q21）、IGH/MAF（14q32/16q23）、IGH/FGFR3（14q32/4p16.3）和IGH/CCND1（14q32/11q13）（美国雅培分子有限公司产品）。每种探针计数200个间期细胞，各探针检测结果异常的阳性阈值以实验室检测阈值为准。

4. 随访：通过电子病案系统收集患者的随访信息。随访截止时间为2023年9月1日，中位随访时间为32（95％ *CI* 30.4～36）个月。OS期定义为从诊断至最后一次随访或死亡的时间。PFS期定义为从患者初始治疗到疾病进展、复发或因任何原因死亡的时间。

5. 统计学处理：采用SPSS 29.0和GraphPad Prism 9.0进行统计学分析。分类变量采用例数（百分比）进行描述，分类变量的组间比较采用Fisher精确检验。连续变量采用*M*（范围）进行描述，非正态分布连续变量的比较采用Mann-Whitney *U*非参数检验。采用Kaplan-Meier法绘制生存曲线，采用Log-rank检验进行组间比较。*P*<0.05为差异有统计学意义。

## 结果

1. 基本特征：564例初治MM及各亚组患者的基线临床特征见表1。19例伴t（14;16）的初治MM患者中男性10例（52.6％），≥65岁7例（36.8％），ISS Ⅲ期13例（68.4％）。M蛋白类型为IgG患者11例（57.9％），HGB<100 g/L患者14例（73.7％）。关于诱导治疗，8例（42.1％）患者采用以PI为基础的方案，11例（57.9％）患者采用PI联合IMiD方案。8例（42.1％）患者接受auto-HSCT。

**表1:** 564例初治多发性骨髓瘤及各亚组患者的基线临床特征

特征	总体（564例）	伴t(14;16)（19例）	FISH正常（191例）	伴t(4;14)（109例）	伴del(17p)（55例）
男性［例（%）］	328（58.2）	10（52.6）	125（65.4）	61（56.0）	24（43.6）
年龄≥65岁［例（%）］	173（30.7）	7（36.8）	51（26.7）	38（34.9）	20（36.4）
ISS分期［例（%）］					
Ⅰ	116（20.6）	3（15.8）	59（30.9）	11（10.1）	12（21.8）
Ⅱ	183（32.4）	3（15.8）	70（36.6）	39（35.8）	14（25.5）
Ⅲ	265（47.0）	13（68.4）	62（32.5）	59（54.1）	29（52.7）
M蛋白类型［例（%）］					
IgG	261（46.3）	11（57.9）	85（44.5）	64（58.7）	24（43.6）
IgA	122（21.6）	3（15.8）	40（20.9）	32（29.4）	13（23.6）
IgD	33（5.9）	0（0）	14（7.3）	1（0.9）	4（7.3）
IgM	1（0.2）	0（0）	0（0）	0（0）	0（0）
κ	71（12.6）	3（15.8）	22（11.5）	8（7.3）	6（10.9）
λ	64（11.3）	2（10.5）	24（12.6）	4（3.7）	5（9.1）
未分泌	12（2.1）	0（0）	6（3.2）	0（0）	3（5.5）
HGB<100 g/L［例（%）］	321（56.9）	14（73.7）	93（48.7）	73（67.0）	33（60.0）
LDH≥ULN［例（%）］	97（17.2）	4（21.1）	27（14.1）	20（18.3）	45（81.8）
白蛋白［g/L，M（范围）］	35.6（14.7～336.0）	38.5（21.8～47.0）	36.6（14.7～129.0）	30.5（17.9～336.0）	36.2（17.9～336.0）
肌酐［µmol/L，M（范围）］	82.6（5.0～1 708.0）	92.0（48.4～359.7）	75.0（22.0～1 708.0）	88.0（35.3～1 213.0）	72.2（33.8～525.2）
血钙≥2.75 mmol/L［例（%）］	64（11.3）	1（6.3）	15（7.9）	17（15.6）	5（9.1）
β_2_-MG≥5.5 mg/L［例（%）］	244（43.3）	11（57.9）	68（35.6）	50（45.9）	24（43.6）
诱导治疗方案［例（%）］					
PI	290（51.4）	8（42.1）	106（55.5）	57（52.3）	19（34.5）
IMiD	37（6.6）	0（0）	21（11.0）	4（3.7）	2（3.6）
PI+IMiD	237（42.0）	11（57.9）	64（33.5）	48（44.0）	34（61.8）
auto-HSCT［例（%）］	225（39.9）	8（42.1）	87（45.5）	47（43.1）	18（32.7）

**注** ULN：正常值上限；β_2_-MG：β_2_-微球蛋白；PI：蛋白酶体抑制剂；IMiD：免疫调节药物

2. 生存分析：564例初治MM患者的中位PFS期为47.3（95％ *CI* 37.9～56.7）个月，中位OS期未达到（NR），3年OS率为71.4％（95％ *CI* 67.1％～74.3％）。

19例伴t（14;16）和191例FISH正常患者中位随访36.0（1.0～70.4）个月，两组患者的中位PFS期分别为14.0（95％ *CI* 4.5～23.5）个月和NR（*P*<0.001），3年的PFS率分别为38.4％（95％ *CI* 16.0％～60.8％）和72.3％（95％ *CI* 64.8％～78.6％），中位OS期分别为42.0（95％ *CI* 15.4～68.6）个月和NR（*P*＝0.002），3年OS率分别为54.8％（95％ *CI* 26.7％～76.1％）和79.5％（95％ *CI* 72.4％～85.0％）（图1）。

**图1:**
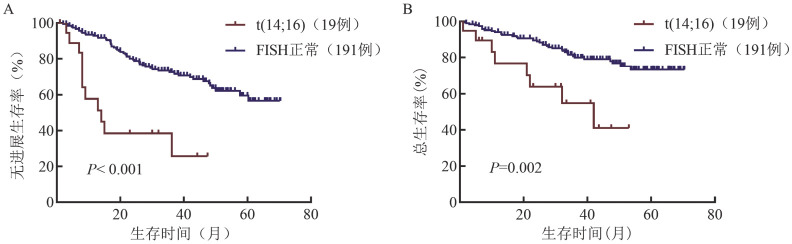
伴t（14;16）和FISH正常多发性骨髓瘤患者的无进展生存（A）和总生存（B）曲线

通过PSM得到15对伴t（14;16）和伴t（4;14）患者，两组患者各基线临床特征的差异均无统计学意义（*P*值均>0.05）。伴t（14;16）和伴t（4;14）患者的中位随访时间为22.0（1.0～47.5）个月，两组患者的中位PFS期分别为13.0（95％ *CI* 2.8～23.1）个月和NR（*P*＝0.247），中位OS期分别为42个月和NR（*P*＝0.609），差异均无统计学意义（图2）。

**图2:**
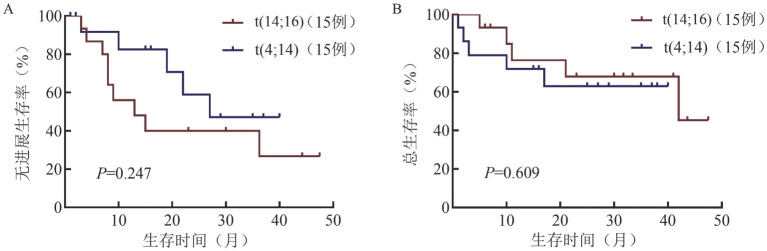
倾向性评分匹配后伴t（14;16）和伴t（4;14）多发性骨髓瘤患者的无进展生存（A）和总生存（B）曲线

通过PSM得到15对伴t（14;16）和伴del（17p）患者，两组患者各基线临床特征的差异均无统计学意义（*P*值均>0.05）。伴t（14;16）和伴del（17p）患者的中位随访时间为26.5（4.0～57.0）个月，两组患者的中位PFS期分别为13.0（95％ *CI* 2.8～23.2）个月和31.0（95％ *CI* 15.7～46.2）个月（*P*＝0.939），中位OS期分别为42个月和37.3（95％ *CI* 31.5～43.1）个月（*P*＝0.557），差异均无统计学意义（图3）。

**图3:**
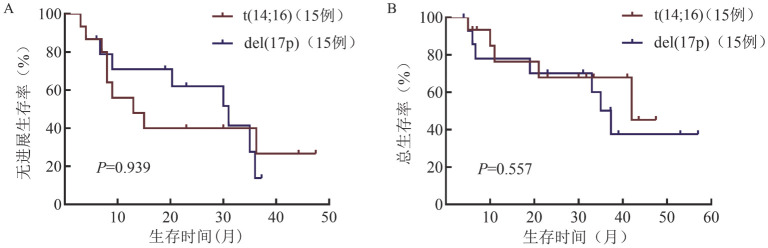
倾向性评分匹配后伴t（14;16）和伴del（17p）多发性骨髓瘤患者的无进展生存（A）和总生存（B）曲线

19例伴t（14;16）患者中14例（73.7％）合并1q21+，109例伴t（4;14）患者中72例（66.1％）合并1q21+，两组的差异无统计学意义（*P*＝0.517）。伴t（14;16）合并1q21+患者和伴t（4;14）合并1q21+患者的中位随访时间为26.0（1.0～69.0）个月，两组的中位PFS期分别为13.0（95％ *CI* 4.7～21.2）个月和30.7（95％ *CI* 14.8～46.6）个月（*P*＝0.102），中位OS期分别为42.0（95％ *CI* 19.5～64.5）个月和56.0（95％ *CI* 10.1～102.0）个月（*P*＝0.888），差异均无统计学意义。通过PSM得到的15对伴t（14;16）和t（4;14）患者中，分别有10例伴t（14;16）患者和12例伴t（4;14）患者合并1q21+，两组各基线临床特征的差异均无统计学意义（*P*值均>0.05），两组患者的中位PFS期分别为9.0（95％ *CI* 1.9～16.1）个月和27.0（95％ *CI* 16.6～37.4）个月（*P*＝0.323），中位OS期分别为NR和42个月（*P*＝0.320），差异均无统计学意义。

55例伴del（17p）患者中33例（60.0％）合并1q21+，与伴t（14;16）合并1q21+患者相比，差异无统计学意义（*P*＝0.292）。伴t（14;16）合并1q21+患者和伴del（17p）合并1q21+患者的中位随访时间为19.0（1.0～57.0）个月，两组的中位PFS期分别为13.0（95％ *CI* 4.7～21.2）个月和27（95％ *CI* 13.1～40.9）个月（*P*＝0.972），中位OS期分别为42.0（95％ *CI* 19.5～64.5）个月和30.0（95％ *CI* 15.6～44.4）个月（*P*＝0.447），差异均无统计学意义。PSM得到的15对伴t（14;16）和伴del（17p）患者中，分别有10例伴t（14;16）患者和7例伴del（17p）患者合并1q21+，两组各基线临床特征的差异均无统计学意义（*P*值均>0.05），两组患者的中位PFS期分别为9.0（95％ *CI* 1.9～16.1）个月和30.0（95％ *CI* 0～62.1）个月（*P*＝0.951），中位OS期分别为NR和33.0（95％ *CI* 3.8～62.2）个月（*P*＝0.437），差异均无统计学意义。

19例伴t（14;16）患者中3例（15.8％）合并del（17p），3例患者的中位PFS期和中位OS期分别为4.0（95％ *CI* 2.4～5.6）个月和NR，3年OS率为66.7％（95％ *CI* 5.4％～94.5％）。

19例伴t（14;16）患者中8例接受auto-HSCT，11例未接受auto-HSCT，19例患者的中位随访时间为23（1～53）个月。接受与未接受auto-HSCT患者的中位PFS期分别为15.0（95％ *CI* 12.4～17.6）个月和8.0（95％ *CI* 7.1～8.9）个月（*P*＝0.291），中位OS期分别为42个月和32.0（95％ *CI* 0～72.7）个月（*P*＝0.328），差异均无统计学意义。

## 讨论

细胞遗传学异常在MM的危险分层中有重要作用。近年来，新药的不断出现极大地改善了新诊断MM（NDMM）患者的预后。但仍有一些伴HRCA的患者预后差，在诊断后2～3年早期死亡[10]。及早识别高危MM患者并应用有效的个体化治疗具有重要意义。t（14;16）在3％～5％的初治MM患者中被发现[2]–[3],[11]，各指南将t（14;16）纳入HRCA[1],[6],[12]。由于较为罕见，t（14;16）的预后价值并不如t（4;14）和del（17p）明确。2009版IMWG未把t（14;16）归为HRCA，R2-ISS也因为t（14;16）样本量少，PFS的差异无统计学意义（*P*＝0.130），没有将其纳入预后评分系统中。本研究分析了国内两个中心564例NDMM患者，其中19例（3.4％）患者具有t（14;16）异常，结果显示，与FISH正常的患者相比，t（14;16）患者具有较短的PFS期和OS期，与既往研究的结果一致[2],[4]。一项法国多中心研究结果显示，与不伴t（14;16）患者相比，伴t（14;16）患者并不具有更差的预后（*P*＝0.280），该研究纳入的患者中60％接受了双次auto-HSCT治疗[13]，有研究显示双次auto-HSCT可能使伴有HRCA的患者获益[14]。法国骨髓瘤工作组（IFM）发表了一项纳入169例t（14;16）患者的大型研究[3]，该研究认为t（14;16）患者的不良预后是由于合并其他HRCA，t（14;16）不会导致预后不良，但仅伴有t（14;16）的例数太少（13例），还需要增加样本量验证。为进一步探索t（14;16）的预后价值，我们按照1:1进行PSM分析，分别比较伴t（14;16）与伴t（4;14）、del（17p）患者的预后差异。结果显示，伴t（14;16）与t（4;14）患者PFS、OS的差异均无统计学意义。伴t（14;16）和del（17p）患者PFS和OS的差异均无统计学意义。一项韩国研究分析了555例MM患者，结果显示，伴del（17p）患者和t（14;16）患者auto-HSCT后的中位PFS期相近，分别为11个月和9个月[15]。以色列的一项研究和本研究结果相近，39例伴t（14;16）患者和99例伴t（4;14）患者的中位OS期分别为3年和4.2年，差异无统计学意义[16]。尽管伴t（14;16）的患者数量少，其预后应当具有与t（4;14）和del（17p）一样的价值，未来有待积累更多数据进一步验证这一结论。

在MM中，APOBEC突变与发生继发性细胞遗传学异常的分子机制相关[17]，而APOBEC突变频率和数目在t（14;16）中最高[18]。目前一项关于t（14;16）的研究指出，近2/3伴t（14;16）的患者伴有其他HRCA，其中伴1q21+患者占81.3％，伴del（17p）患者占21.5％[19]。另一项大型研究显示，伴t（14;16）患者较不伴t（14;16）患者更多地合并1q21+（69.2％对29.1％，*P*<0.001）和del（17p）（22.5％对8.7％，*P*<0.001）[3]。本研究结果显示，伴t（14;16）的19例患者中14例伴1q21+（73.7％），与t（4;14）伴1q21+患者（66.1％）及del（17p）伴1q21+（60.0％）相比，差异均无统计学意义（*P*值均>0.05）。生存分析显示，对于合并1q21+的患者，t（14;16）的预后与t（4;14）及del（17p）相比无明显差异。国内一项关于1q21+患者预后危险分层系统的研究显示，t（14;16）是一个独立危险因素，伴1q21+的t（14;16）患者预后更差[20]。上述研究与本研究结果存在差异的原因可能与治疗方式和患者的异质性相关。虽然t（14;16）合并del（17p）患者的病例数少（3例，15.8％），仍然表现出较短的PFS期（中位PFS期为4个月）。t（14;16）合并1q21+和del（17p）患者仍需要更强的化疗方案以改善预后。

尽管auto-HSCT能显著延长MM患者的生存期，一些研究表明，对于伴有HRCA的患者来说，auto-HSCT的作用有限[7]。本研究显示，接受和未接受auto-HSCT的t（14;16）患者的PFS和OS期差异均无统计学意义，说明auto-HSCT并不能改善t（14;16）患者的生存。Narita等[4]的研究显示，41例接受auto-HSCT患者中，伴t（14;16）患者（8例）的PFS期明显短于不伴t（14;16）的患者（33例）（*P*＝0.031），与本研究结果一致。一项单臂、Ⅱ期MASTER试验结果显示，达雷妥尤单抗+卡非佐米+来那度胺+地塞米松联合auto-HSCT治疗可使HRCA数量≤1的NDMM患者获得较好的预后，但对于HRCA数量≥2的NDMM患者的疗效则不明显[21]。本研究中大部分伴t（14;16）患者合并其他HRCA，即使接受auto-HSCT也不能从中获益。也许未来c-MAF可作为伴t（14;16）的MM患者的潜在治疗靶点[22]–[23]。

总之，t（14;16）在初治MM中属于较为少见的细胞遗传学异常，其预后与伴t（4;14）及del（17p）患者无明显差异。伴t（14;16）患者常合并其他HRCA，预后更差，auto-HSCT不能改善t（14;16）患者的不良预后，未来需要探索更多新的治疗方式。

## References

[1] Rajkumar SV (2024). Multiple myeloma: 2024 update on diagnosis, risk-stratification, and management[J]. Am J Hematol.

[2] Liu Y, Lv R, Yan W (2024). MAF translocation remains a strong prognostic factor despite concurrent chromosomal abnormalities[J]. Haematologica.

[3] Schavgoulidze A, Perrot A, Cazaubiel T (2023). Prognostic impact of translocation t(14;16) in multiple myeloma according to the presence of additional genetic lesions[J]. Blood Cancer J.

[4] Narita T, Inagaki A, Kobayashi T (2015). t(14;16)-positive multiple myeloma shows negativity for CD56 expression and unfavorable outcome even in the era of novel drugs[J]. Blood Cancer J.

[5] Hanamura I (2022). Multiple myeloma with high-risk cytogenetics and its treatment approach[J]. Int J Hematol.

[6] Palumbo A, Avet-Loiseau H, Oliva S (2015). Revised International Staging System for Multiple Myeloma: A Report From International Myeloma Working Group[J]. J Clin Oncol.

[7] Sonneveld P, Avet-Loiseau H, Lonial S (2016). Treatment of multiple myeloma with high-risk cytogenetics: a consensus of the International Myeloma Working Group[J]. Blood.

[8] Munshi NC, Anderson KC, Bergsagel PL (2011). Consensus recommendations for risk stratification in multiple myeloma: report of the International Myeloma Workshop Consensus Panel 2[J]. Blood.

[9] D'Agostino M, Cairns DA, Lahuerta JJ (2022). Second Revision of the International Staging System (R2-ISS) for Overall Survival in Multiple Myeloma: A European Myeloma Network (EMN) Report Within the HARMONY Project[J]. J Clin Oncol.

[10] 中国抗癌协会血液肿瘤专业委员会骨髓瘤与浆细胞疾病学组, 中国临床肿瘤学会多发性骨髓瘤专家委员会 (2024). 高危多发性骨髓瘤诊断与治疗中国专家共识(2024年版)[J]. 中华血液学杂志.

[11] Mina R, Joseph NS, Gay F (2020). Clinical features and survival of multiple myeloma patients harboring t(14;16) in the era of novel agents[J]. Blood Cancer J.

[12] Touzeau C, Perrot A, Hulin C (2024). Daratumumab, carfilzomib, lenalidomide, and dexamethasone with tandem transplant for high-risk newly diagnosed myeloma[J]. Blood.

[13] Avet-Loiseau H, Malard F, Campion L (2011). Translocation t(14;16) and multiple myeloma: is it really an independent prognostic factor?[J]. Blood.

[14] Kumar SK, Callander NS, Adekola K (2023). Multiple Myeloma, Version 2.2024, NCCN Clinical Practice Guidelines in Oncology[J]. J Natl Compr Canc Netw.

[15] Byun JM, Shin DY, Hong J (2018). Distinct predictive impact of FISH abnormality in proteasome inhibitors and immunomodulatory agents response: redefining high-risk multiple myeloma in Asian patients[J]. Cancer Med.

[16] Duek A, Trakhtenbrot L, Amariglio N (2019). Newly diagnosed multiple myeloma patients carrying monoallelic deletion of the whole locus of immunoglobulin heavy chain gene have a better prognosis compared to those with t(4;14) and t(14;16)[J]. Genes Chromosomes Cancer.

[17] Ziccheddu B, Giannotta C, D'Agostino M (2024). Genomic and immune determinants of resistance to daratumumab-based therapy in relapsed refractory multiple myeloma[J]. Blood Cancer J.

[18] Hoang PH, Cornish AJ, Dobbins SE (2019). Mutational processes contributing to the development of multiple myeloma[J]. Blood Cancer J.

[19] Goldman-Mazur S, Jurczyszyn A, Castillo JJ (2020). A multicenter retrospective study of 223 patients with t(14;16) in multiple myeloma[J]. Am J Hematol.

[20] Yang P, Chen H, Liang X (2023). Proposed risk-scoring model for estimating the prognostic impact of 1q gain in patients with newly diagnosed multiple myeloma[J]. Am J Hematol.

[21] Costa LJ, Chhabra S, Medvedova E (2022). Daratumumab, Carfilzomib, Lenalidomide, and Dexamethasone With Minimal Residual Disease Response-Adapted Therapy in Newly Diagnosed Multiple Myeloma[J]. J Clin Oncol.

[22] Mian H, Kaiser M, Fonseca R (2024). Still high risk? A review of translocation t(14;16) in multiple myeloma[J]. Am J Hematol.

[23] Deng Y, Lu L, Zhang H (2023). The role and regulation of Maf proteins in cancer[J]. Biomark Res.

